# Influence of Anchoring on Burial Depth of Submarine Pipelines

**DOI:** 10.1371/journal.pone.0154954

**Published:** 2016-05-11

**Authors:** Yuan Zhuang, Yang Li, Wei Su

**Affiliations:** School of Navigation, Wuhan University of Technology, Wuhan, Hubei, China; Seoul National University, REPUBLIC OF KOREA

## Abstract

Since the beginning of the twenty-first century, there has been widespread construction of submarine oil-gas transmission pipelines due to an increase in offshore oil exploration. Vessel anchoring operations are causing more damage to submarine pipelines due to shipping transportation also increasing. Therefore, it is essential that the influence of anchoring on the required burial depth of submarine pipelines is determined. In this paper, mathematical models for ordinary anchoring and emergency anchoring have been established to derive an anchor impact energy equation for each condition. The required effective burial depth for submarine pipelines has then been calculated via an energy absorption equation for the protection layer covering the submarine pipelines. Finally, the results of the model calculation have been verified by accident case analysis, and the impact of the anchoring height, anchoring water depth and the anchor weight on the required burial depth of submarine pipelines has been further analyzed.

## Introduction

Due to an increase in exploration of domestic marine oil and gas resources, there have been more submarine pipelines constructed which is resulting in more obvious damage due to ship anchoring operations [1–3]. Many countries are now strengthening research in this field to better protect submarine pipelines [4].

Studies on damage to submarine pipelines due to anchoring has been active in many countries since the late 1950s [5–7]. Several related specifications have been established, such as the ISO standard, Australian national standards, Canadian national standards, Japanese national standards, etc. As submarine pipeline damage is still occasionally occurring globally, many countries have strengthened their research in this area in order to better protect the safety of their submarine pipelines. Currently, researchers in Norway and Japan are leading in this area, and the research of Det Norske Veritas (DNV) on protection technology for submarine pipelines is at an internationally advanced level [8]. By undertaking systematic tests and studies, DNV have made large-scale modifications to the specifications for offshore engineering operations and created specifications for risk assessment of submarine pipeline protection in 2001, referred to as DNV-RP-F107 [9]. These specifications have corresponding technical guidance for the development of off-shore oilfields as well as guidance for protecting submarine pipelines [10, 11]. However, US legislation, which is focused on the Gulf of Mexico, includes detailed and specific provisions relating to the digging and burial depth of submarine pipelines within its own territory, making it the most valuable reference of all legislations and specifications related to digging and burial depth [12].

Mousselli et al. have highlighted the importance of the mechanics of anchor-soil interactions and of the capacity of the pipe to absorb energy [5], while Hvam et al. have discussed the main parameters relevant to assessment of the risk of pipe damage from dragging ship anchors, in particular the frequency of pipe-anchor interactions and of pipe damage [6].Alexander et al. have used testing methods to evaluate anchor impact damage to subsea canyon chief pipelines [13]. Putranta et al. have focused on assessment of the risk to gas pipelines due to dredging activities, including risk assessment during operation of a jetty after dredging [14]. In Srisk and arajah’s paper, Finite Element Analysis has been used to assess the integrity of a pipeline against the dynamic effects due to interactions with ships’ anchors, and have investigated the potential for mechanical damage to a pipeline based on the effects of various vessel and anchor configurations [15]. Rességuier et al. have performed calculations to assess the penetration of trawl boards of different sizes, weights and a range of soil strengths, and have confirmed that the penetration of trawl gear is very limited for most clays, with the exception of very soft clay where excessive penetration can be expected [16].

A lot of researchers have conducted studies on trenching and burial depth of submarine pipelines. Wang Zaiming has proposed an impact probability model that describes the probability of impact between anchors and submarine pipelines under different anchoring conditions based on the basic principles of probability theory [17]. However, his research lacks consideration of the penetration depth of the anchor. His research includes particular reference values for further development of a digging and burial depth model for submarine pipelines from the perspective of risk theory. Liu Huan et al. have established an energy calculation model for calculating the direct positive impact on submarine pipelines during an anchoring operation and have presented a judgment method for evaluation of the damage level of the submarine pipelines. They have studied the correlation coefficients for laying submarine pipelines in detail, which has provided a theoretical basis for further studies on the basis of a risk evaluation method for submarine pipelines and with reference to probability theory and other theories [18]. Tan Jian et al. have optimized the probability model and studied the damage caused by anchors impacting on submarine pipelines. They have conducted a corresponding study on the damage level of submarine pipelines under impact and have proposed corresponding protection regulations for submarine pipelines [19]. To address emergency anchoring operations, Zhang Lei et al. have studied the effect of emergency anchoring operations on the required burial depth of submarine pipelines and the effect of drag-anchor sailing on the submarine pipelines systematically. They have proposed an optimized scheme for the rap ripping protection layer [20].

There are still several problems that exist, necessitating further research into the required burial depth of submarine pipelines and the strain mechanisms of anchor damage, including: 1. The burial depth requirements for protection of submarine pipelines have been established from practical experience but require further validation from a theoretical and experimental basis; additionally, technical specifications or standards with a broader applicability have not yet been formed. 2. Analysis of forces during the anchor dropping phase has not been very systematic or comprehensive. The extent that these forces affect the anchor speed calculations when an anchor penetrates the seabed and the final geological penetration depth should be investigated. 3. Currently, studies only calculate and solve the problem of the penetration depth of the anchor under normal circumstances. There is a lack of generalized study, analysis and calculation on the vertical penetration depth of anchors with different weights under conditions of different water depths and different sediments.

Based on previous research, this paper uses practical experience of ship handling to study the overall process of anchoring operations. For each of the different methods of anchoring, mathematical models for ordinary anchoring and deep water anchoring have been established to derive an equation for the anchor impact energy for each set of conditions. Determination of the energy of the covering layer depends on the energy of the anchor and the energy absorption ability of the submarine pipeline. Therefore, the required thickness of the protective layer has been calculated using a formula for the covering layer absorption energy. Finally, the weight of the anchor, the depth and the height of the anchoring operations have been analyzed and it has been confirmed that the mass of the anchor has the greatest influence on the required burial depth of the submarine pipeline, while the height and depth have a smaller influence. We hope that this article will provide a theoretical basis for future research in the field of offshore pipeline burial depth.

## Model Formulation Based on Anchoring Operations

Different anchoring methods are generally employed depending on the water depth. In this section, mathematical models will be established for two situations, namely ordinary anchoring and deep water anchoring.

### Model formulation based on ordinary anchoring

**Fig 1:**
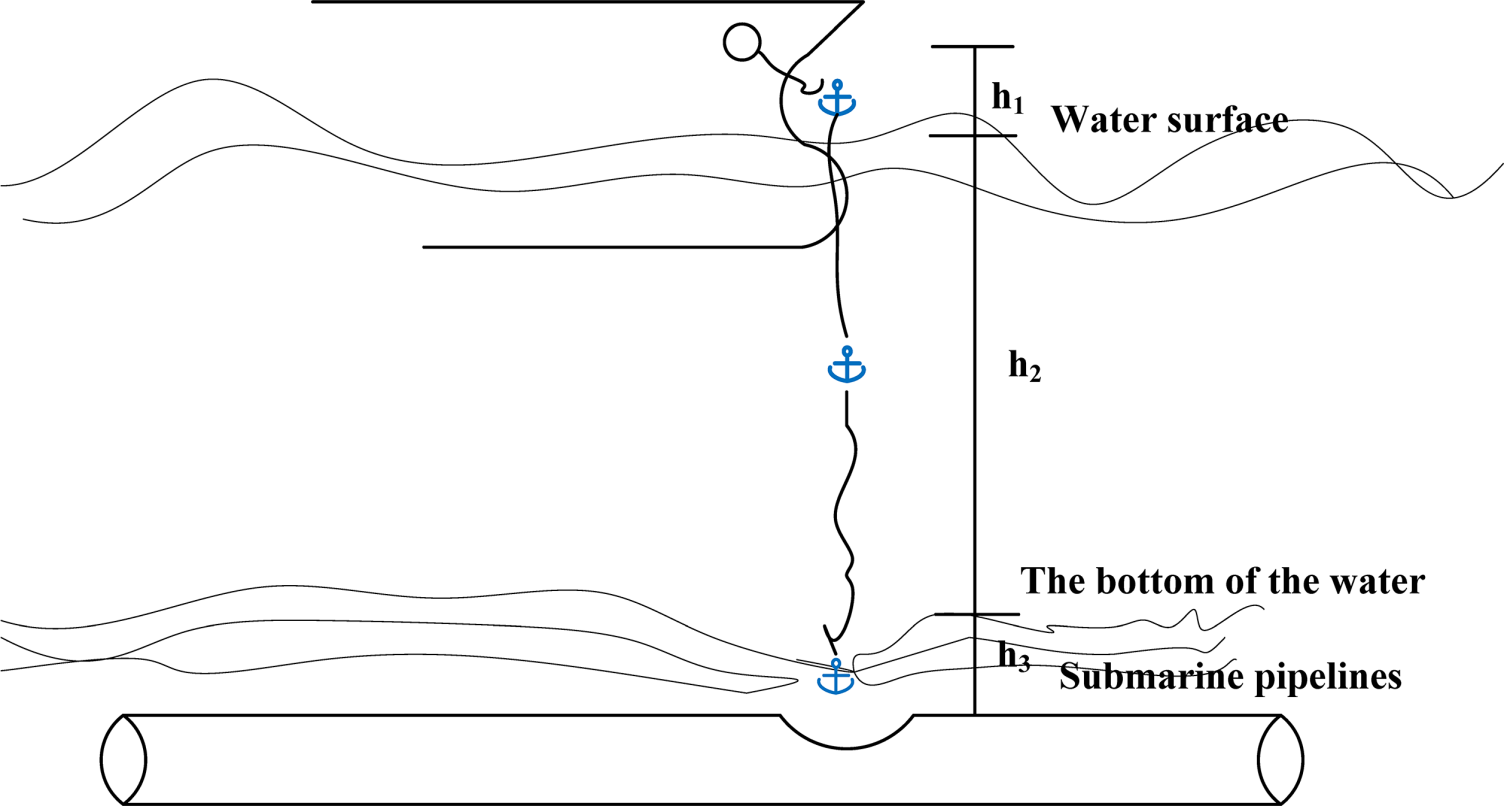
Schematic diagram of falling movement of the anchor for ordinary anchoring. *h*_*1*_- height of the anchor-dropping position above the water surface, m; *h*_*2*_- height of water surface above the bottom of the water, m; *h*_*3*_- depth that the anchor penetrates into the seabed, m.

To clearly analyze anchor movement during ordinary anchoring, the process is separated into three stages [19], with a movement diagram; see Fig 1.

#### From dropping the anchor to the moment that the anchor reaches the water surface

Under ideal conditions, when an anchor is dropped, it travels with constant acceleration until it reaches the water. However, it may be affected by factors such as wind power. In this paper, an anchor movement model [21] under ideal conditions has been established, where the anchor windlass effect was not taken into consideration. During this stage, the anchor experiences downward acceleration due to gravity from its weight [22]. Air resistance is not included so gravity is the only factor influencing the anchor, as shown below:

$$mgh_{1}=\frac{1}{2}mv_{1}{}^{2}-\frac{1}{2}mv_{0}^{2}$$ (1)

The anchor velocity when it first touches the water surface can be expressed by the following equation:

$$v_{1}=\sqrt{2gh_{1}}$$ (2)

Where: *m*- weight of the anchor, kg; *v*_*0*_- initial velocity of the dropped anchor, 0; *v*_*1*_- velocity of the anchor when touching the water, m/s.

#### From the moment the anchor reaches the water surface until the anchor reaches the bottom of the water

While in the water, the anchor is subject to self-gravity, water resistance and the tension force of the anchor chain. The anchor’s movement is determined from a combination of these three factors. The water depth has a major influence on the anchor velocity. The anchor impact energy before it reaches the seabed floor will depend on its own weight and velocity [23].

From falling into the water until it reaches the floor, the anchor is subject to gravity *mg*, a buoyant force *B* and fluid resistance *f*. Using a small Reynolds number Re for fluid resistance (the criterion for fluid flow pattern), the acting forces on the anchor under the water are:

mg-B-f=mdv/dt (3)

Buoyant force:B=ρVg (4)

where: *ρ* - mass density of the sea water, *kg/m*^*3*^;*v*- velocity of the dropping anchor, *m/s*; *f* - viscous resistance of the anchor, *N*; *B*- buoyant force of the anchor, *N*; *g*- gravitational acceleration, *m/s*^*2*^;*V*- volume of the anchor, *m*^*3*^.

When the anchor falls freely in water, it is subject to water resistance, with a force in the opposite direction to the direction of falling. Water resistance depends on factors such as the shape, size and velocity of the anchor, the sea water temperature, viscosity and the density coefficient. Water resistance is proportional to velocity when an object is moving at relatively low speeds [24][25].

The water resistance of the anchor can be expressed as follows:

f=kv=6πηrv (5)

where: *f*- viscous resistance of the anchor, *N*; *v*- velocity of the dropping anchor, *m/s*; *r*- effective radius of the anchor, *m*; *v*_*2*_- anchor velocity when it reaches the bottom, *m/s*; *η* - viscosity coefficient of water, 1.002×10^3^ *N/m*^*2*^ when the sea water temperature is 20 degree Celsius.

Under normal conditions (1 atmosphere, 20 degrees Celsius), the stress state of the ship is determined by Eqs (3) and (5). Combining the two equations:

$$mg-B-kv\;=m\frac{dv}{dt}$$ (6)

Divide both sides by k:

$$\frac{mg-B}{k}-v=\frac{m}{k}\cdot \frac{dv}{dt}$$ (7)

Let $K=\frac{mg-B}{k}$, then:

$$\frac{dv}{v\;-K}=-\frac{k}{m}\cdot dt$$ (8)

Compute the integral for the above equation:

$$\int_{v_{1}}^{v}\frac{dv}{v-K}=\int_{0}^{t}-\frac{k}{m}dt$$ (9)

Thus:

$$\text{ln}(v-K)-\text{ln}(v_{1}-K)=-\frac{k}{m}t$$ (10)

Then:

$$v\;=K+(v_{1}-K)\cdot e^{-\frac{k}{m}\cdot t}$$ (11)

While *v* = *v*_2_, *t* = *T*, then:

$$v_{2}=K+(v_{1}-K)\cdot e^{-\frac{k}{m}\cdot T}$$ (12)

Since $v=\frac{dx}{dt}$, i.e. *dx* = *vdt*, then:

$$\int_{0}^{h_{2}}dx=\int_{0}^{T}\left[K+(v_{1}-K\right)\cdot e^{-\frac{k}{m}t}]dt$$ (13)

Thus:

$$h_{2}=\frac{mg-B}{k}T+\frac{mg-B}{k}\cdot \frac{m}{k}(e^{-\frac{k}{m}T}-1)-\frac{m}{k}\sqrt{2gh_{1}}(e^{-\frac{k}{m}T}-1)$$ (14)

The falling time T of the anchor in water can be obtained by solving Eq (14). Then the falling time T can be substituted into Eq (12) to derive the velocity *v*_*2*_. Therefore, with regards to *v*_*2*_ and the anchor weight *m*, the energy of the impact on the submarine pipeline can be determined for the ordinary anchor operation [26]:

$$Q=mv_{2}{}^{2}/2$$ (15)

#### From the moment the anchor penetrates the floor until it stabilizes

The anchor impacts the sand on the seabed floor with huge energy and penetrates through a certain thickness of the soil sand layer. During this process, if a submarine pipeline is buried just under the soil sand layer, the anchor may directly hit the pipeline after penetrating the soil sand layer or squeeze the pipeline by striking its protection layer, thereby causing deformation or damage to the submarine pipeline [20].

### Model formulation based on the anchoring operation in deep water

**Fig 2:**
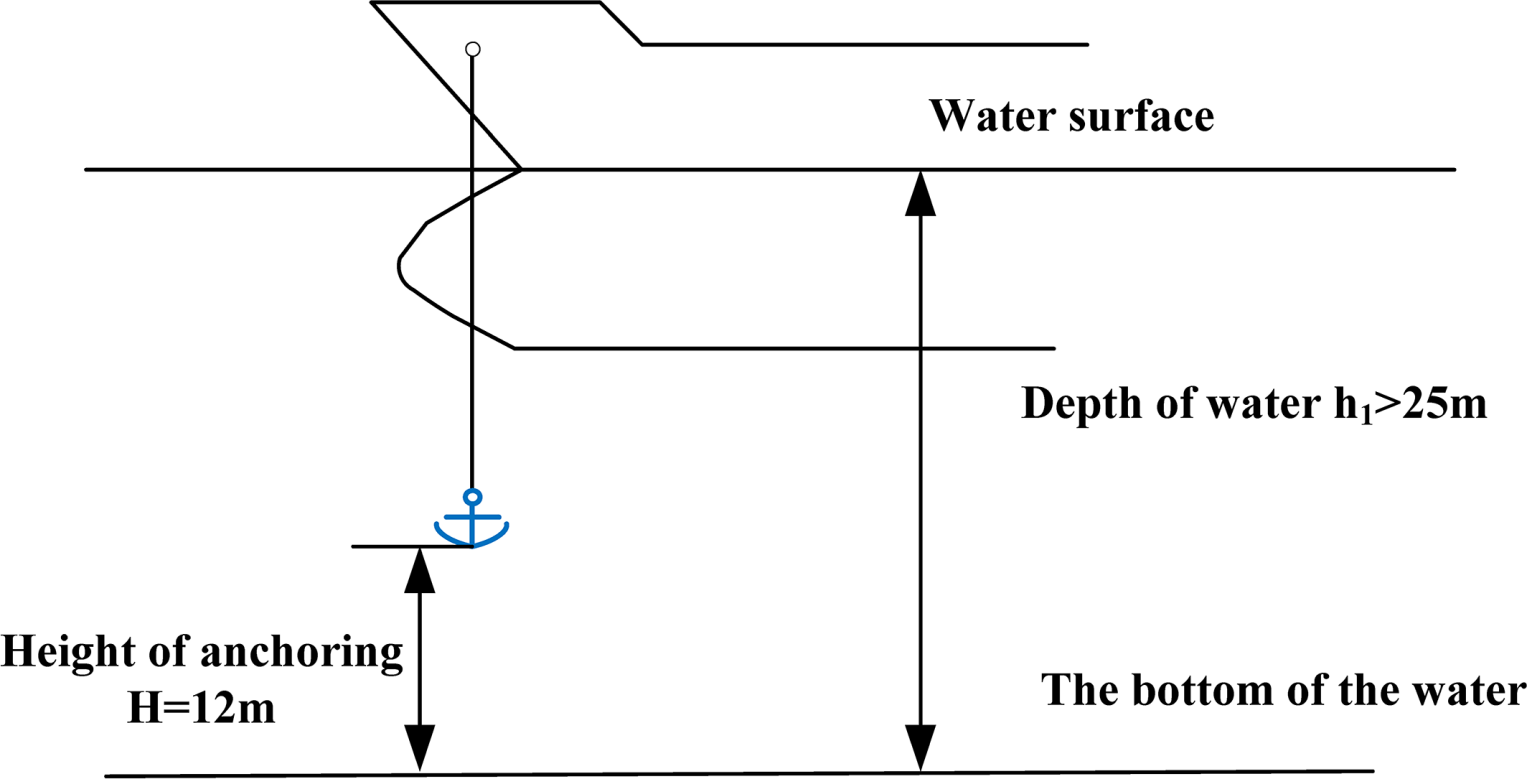
Schematic diagram of falling movement of the anchor during a deep water anchoring operation.

When the depth of water is greater than 25m, deep water anchoring is always adopted to ensure operational safety, where the anchor windlass transports the anchor to 12m from the bottom of the water and then drops it, see Fig 2. If the water depth is more than 50~80m, the anchor windlass is required to cast the anchor to the bottom of the seabed floor. Deep water anchoring can effectively prevent accidents such as the chain breaking or loss of the anchor [26, 27].

When the deep water anchoring method is applied, the anchor is under the influence of gravity, buoyant force and water resistance, and the anchor movement can be expressed as follows, which is similar to ordinary anchoring:

$$mg-B-kv\;=m\frac{dv}{dt}$$ (16)

Then:

$$v\;=K-K\cdot e^{-\frac{k}{m}\cdot t}$$ (17)

$$\text{Where}:\;K=\frac{mg-B}{k}$$

$$h\;=KT+K\cdot \frac{m}{k}(e^{-\frac{k}{m}\cdot T}-1)$$ (18)

For *h* = 12m, the falling time T of the anchor in water can be obtained by solving Eq (18). The falling time T can then be substituted into Eq (17) to derive the velocity *v*_*3*_. Therefore, with regards to *v*_*2*_ and the anchor weight *m*, the energy of the impact on the submarine pipeline can be determined for deep water anchoring operation by:

$$Q=mv_{3}{}^{2}/2$$ (19)

### Calculations on depth of protection layer for submarine pipelines

During installation of submarine pipelines, backfilling and burial with sandstone are usually employed to protect the pipelines. However, when the pipelines’ protection material is constructed from stones and sandy soil mixed together, it can be complex to determine its energy absorption. In this paper, the following energy absorption equation for the backfilling material of the submarine pipelines is adopted [8, 10, 12, 28, 29]:

$$E_{P}=0.5\cdot r'\cdot D\cdot N_{r}\cdot A_{p}\cdot z+r'\cdot N_{q}\cdot A_{p}\cdot z^{2}$$ (20)

where:

*E*_*p*_- energy absorbed by the filling material, *J*;

*r*' - effective volume-weight of the filling material per unit weight, i.e. density per unit volume, *kg/m*^*3*^;

*D*- diameter of submarine pipelines, *m*;

*A*_*p*_- impact area of submarine pipelines, *J/m*^*2*^;

*z*- penetration depth of the falling object through the protection layer, *m*;

*N*_*r*_ *and N*_*q*_- bearing capacity coefficient of the filling material.

The effective burial depth of the protective layer is taken to be the required penetration depth for the filling substance in the pipeline to absorb the impact energy thoroughly, namely:

$$Q=E_{P}$$ (21)

A reasonable pipeline burial depth for ordinary retreat anchoring can be obtained by solving the simultaneous Eqs (15), (20) and (21) as *H*_1_ = *z*_1_. Using the same principles and approach, a reasonable pipeline burial depth for deep water anchoring can also be obtained by solving the simultaneous Eqs (19), (20) and (21) as *H*_2_ = *z*_2_.

A manual sandstone burial method is employed to protect submarine pipelines and effectively prevent their damage due to anchor-dropping and other falling objects. It is possible that the burial depth used during the actual construction process may not be sufficient, or that the construction site draws on local resources and uses sediment backfilling to bury the pipelines. The thickness of the burial layer for submarine pipelines depends on the weight of passing ships, the sea water depth and the material used for the protection layer [24].

The anchor is subject to strong water resistance when falling due to its significant volume, but it is difficult to determine precise value for water resistance due to the anchor’s shape. Since the main part of the anchor fluke is shaped like an arc, the anchor will herein be assumed to be spherical [30, 31].

The bottom part of the anchor, with length L and width B, experiences the highest water resistance during the falling process. According to specifications, the length-width ratio of the anchor bottom is determined from *λ* = L/B = 2.2~2.8. The area affected by water resistance is then shown to be *S*_1_ = *LB* = *L*^2^ / *λ*. Therefore, to effectively protect the pipelines, the minimum value of the water resistance should be considered and the area related to the water resistance should also be minimized i.e. *λ* = 2.8.

The anchor can be considered to be a sphere with an area of *S* = 4*πr*^2^, which is equal to the water resistance area: *S* = 4*πr*^2^ = *S*_1_ = *L*^2^ / 3. Accordingly, the optimized effective radius of the anchor is *r*^2^ = *L*^2^ / 11.2*π* ≈ *L*^2^ / 35, i.e. *r* = *L* / 6. Therefore, an effective radius of *L/6* will be used for all further calculations [32].

## Case Study

**Fig 3:**
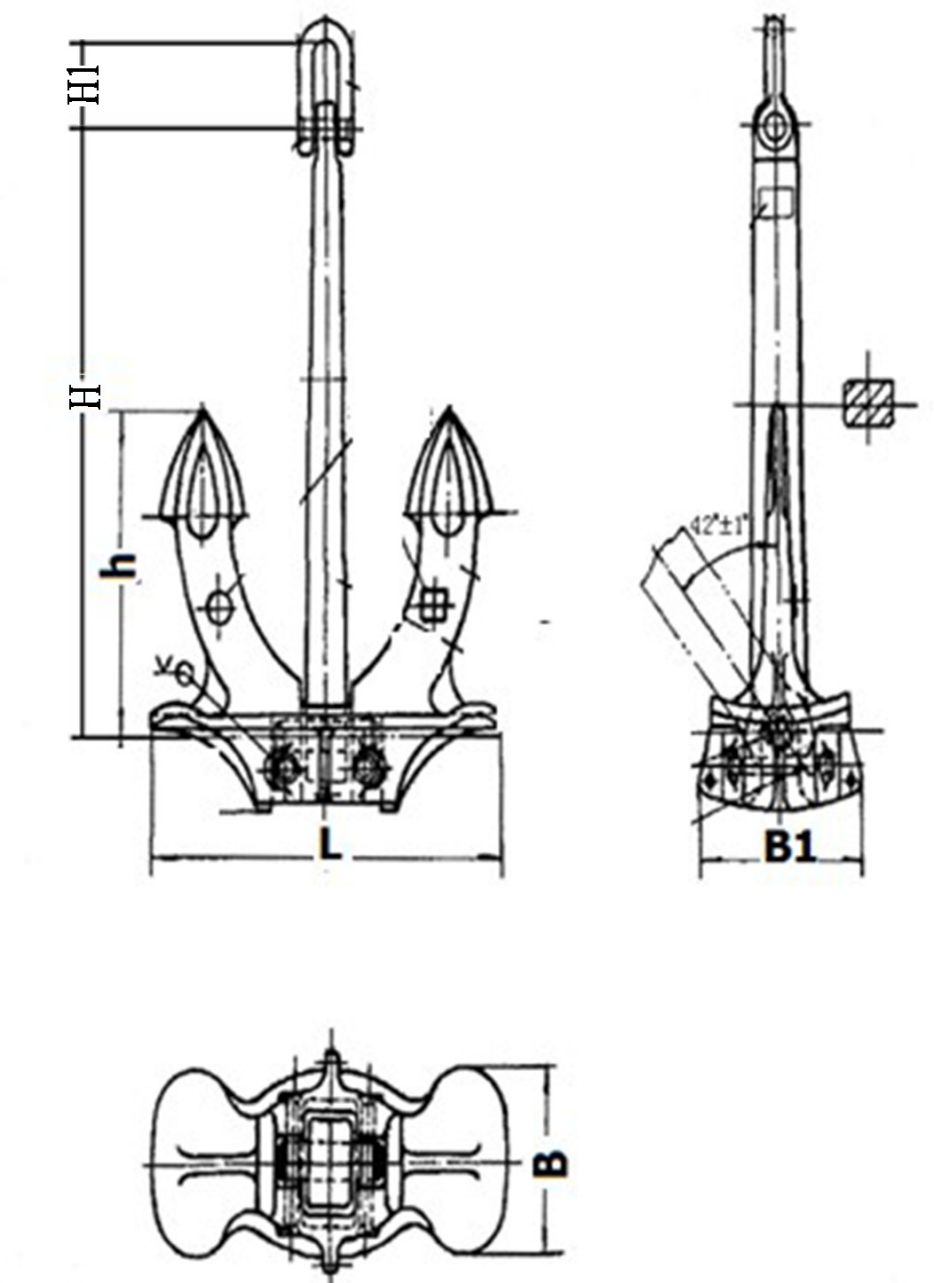
Component names and size of an A type hall anchor. h- length of fluke; H- length of the anchor arm; B1- width of the anchor arm; H1- length of anchor shackle; L- length of the anchor bottom; and B- width of the anchor bottom.

For the representative vessel type, an A type hall anchor, Fig 3, is generally selected for the bow anchor, with size and component names listed in Table 1 [33, 34], according to the national Chinese standards for a “Hall anchor” [35].

**Table 1:** Anchor weights and sizes.

Mass of anchor(kg)	H (mm)	B1 (mm)	H1 (mm)	L (mm)	H (mm)
3060	1283	841	380	1832	2374
4890	1498	984	415	2135	2769
6900	1681	1105	480	2391	3100
10500	1934	1273	600	2752	3571
14100	2135	1404	660	3036	3939
20000	2399	1578	730	3411	4420

For submarine pipelines with a diameter of 273.1mm, a wall thickness of 11.1mm and a yield stress of 530 MPa, a value of 11×10^3^ *kg/m*^*3*^ is the effective volume-weight per unit for the filling material. The bearing capacity coefficients of the filling materials *N*_*r*_ and *N*_*q*_ are set as 137 and 99, for the backward anchoring method, when anchors with various tonnages are freely dropped from a 2m high hawsehole.

### Ordinary anchoring

The falling process of a 3060 kg hall anchor from an anchor drill hole with a height of 2m into water of depth 20m has been analyzed as per the following example:

Set *m* = 3060kg, *L* = 1.832m, *η* = 1.022*10^3^N / m^2^, *h*_1_ = 2m and *h*_2_ = 20m.

Based on $mgh_{1}=\frac{1}{2}mv_{1}{}^{2}-\frac{1}{2}mv_{0}^{2}$, the speed when the anchor just makes contact with the water surface is:

$$v_{1}=\sqrt{2gh_{1}}=\sqrt{2\;*\;9.8\;*\;2}=6.26m/\text{s}$$

The buoyancy of the anchor can be obtained from Eq (4) as:

$$B=\rho Vg=1.03\;*\;10^{3}\;*\;\frac{4}{3}\;*\;\pi \;*\;{\left(\frac{1}{6}\;*\;1.83\right)}^{3}\;*\;9.8=1202.97N,$$

The resistance coefficient is:

$$k=6\pi \eta r=6\pi \;*\;1.022\;*\;1000\;*\;\frac{1}{6}\;*\;1.83=5879.01,$$

$$K=\frac{mg-B}{k}=\frac{3060\;*\;9.8-1202.97}{5879.01}=4.90$$

Substitute the parameters into Eq (14) to obtain:

$$20=4.9\;*\;T+4.9\;*\;\frac{3060}{5879.01}\;*\;(e^{-\frac{5879.01}{3060}\;*\;T}\;-\;1)\;-\;\frac{3060}{5879.01}\;*\;6.26\;*\;(e^{-\frac{5879.01}{3060}\;*\;T}\;-\;1)$$

The time taken for the anchor to make contact with the submarine pipelines is:

T=1.98s

According to Eq (12):

$$v_{2}=K+(v_{1}-K)\cdot e^{-\frac{k}{m}\cdot T}=4.90+(6.26\;-\;4.90)\;*\;e^{-\frac{5879.01}{3060}*1.98}=4.93m/s$$

i.e., the impact speed of a 3060kg Hall anchor thrown freely from a 2m anchor drill hole into 20 m water depth when it hits the submarine pipeline is 4.93m/s.

The impact energy can be expressed as

$$Q=mv_{2}{}^{2}/2=3060\;*\;4.93^{2}/2=37136\text{KJ}$$

Solve the simultaneous Eqs (15), (20) and (21), and substitute the parameters to obtain:

$$37136=0.5*11\;*\;10^{3}*0.2731*137*0.059*{\text{z}}_{1}+11\;*\;10^{3}\;*99*0.059*{\text{z}}_{1}{}^{2}$$

This can be simplified into:

$$37136=12141{\text{z}}_{1}+64251{\text{z}}_{1}{}^{2}$$

The solution is:

$$H_{1}=z_{1}=0.67\text{m}$$

This approach was used to calculate the impact energy and the corresponding reasonable burial depth of submarine pipelines when an anchor falls from 2m height into water depths of 10m, 15m and 20m, respectively. The detailed results are listed in Table 2.

**Table 2:** Required burial depth for submarine pipelines for protection from ordinary anchoring.

Mass of anchor (kg)	Water depth 10m		Water depth 15m		Water depth 20m	
	Impact energy (J)	Burial depth (m)	Impact energy (J)	Burial depth (m)	Impact energy (J)	Burial depth (m)
3060	37211	0.67	36754	0.67	37136	0.67
4890	108557	1.21	109688	1.22	110085	1.22
6900	219750	1.76	233150	1.81	239958	1.84
10500	467887	2.61	530513	2.78	571702	2.89
14100	753006	3.33	889713	3.63	990167	3.83
20000	1263931	4.34	1554392	4.83	1787562	5.18

### Deep water anchoring

A deep water anchoring process using a 3060 kg Hall anchor lowered into a position 12m above the seabed was analyzed as an example.

Deep anchoring is a special type of ordinary anchoring and uses the same calculation principle. The following parameters were used in the calculations:*m* = 3060kg, *L* = 1.832m, *η* = 1.022*10^3^N / m^2^, *h*_1_ = 0m, *h*_2_ = 12m.

Therefore:

$$v_{1}=\sqrt{2gh_{1}}=\sqrt{2\;*\;9.8\;*\;0}=0m/\text{s}$$

According to Section 3.1:

B=1202.97N

k=6πηr=5879.01

$$K=\frac{mg-B}{k}=4.90$$

Substitute the parameters into Eq (18) to obtain:

$$h\;=KT+K\cdot \frac{m}{k}(e^{-\frac{k}{m}\cdot T}-1)$$

$$12=4.9\;*\;T+4.9\;*\;\frac{3060}{5879.01}\;*\;(e^{-\frac{5879.01}{3060}\;*\;T}-1)$$

The time taken for the anchor to make contact with the submarine pipelines is:

T=2.97s

According to Eq (17) $v\;=K-K\cdot e^{-\frac{k}{m}\cdot t}$, giving:

$$v_{2}=K-K\cdot e^{-\frac{k}{m}\cdot T}=4.90\;-\;4.90*\;e^{-\frac{5879.01}{3060}*2.97}=4.88m/s$$

i.e., when a 3060kg Hall anchor is lowered into a position 12m above the seabed, the impact speed of the anchor onto the seabed is 4.88m/s.

The impact energy is:

$$Q=mv_{2}{}^{2}/2=3060\;*\;4.88^{2}/2=36435\text{KJ}$$

Solve the simultaneous Eqs (19), (20) and (21) and substitute the parameters to simplify the equation as:

$$36435=12141{\text{z}}_{2}+64251{\text{z}}_{2}{}^{2}$$

Solve:

$$H_{2}=z_{2}=0.67\text{m}$$

The same approach was used to calculate the impact energy and the corresponding required burial depth of the submarine pipes for deep anchoring of anchors with different weights. The detailed results are listed in Table 3.

**Table 3:** Required burial depth for submarine pipelines so that they are protected from deep water anchoring.

Mass of anchor (kg)	Impact energy (J)	Effective burial depth (m)
3060	36435	0.66
4890	103557	1.18
6900	208032	1.71
10500	443709	2.54
14100	717386	3.25
20000	1211871	4.25

## Discussion

**Fig 4:**
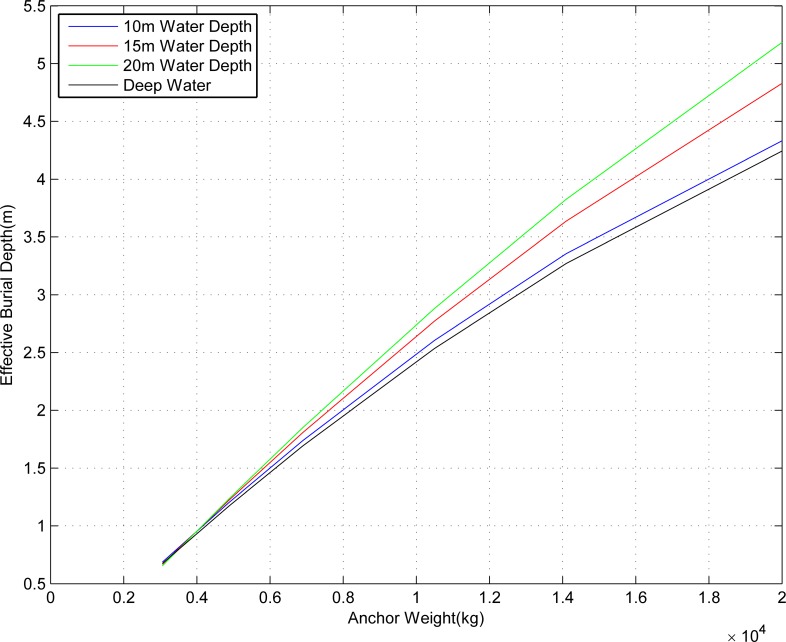
Variation in influences on effective burial depth of submarine pipelines for ordinary anchoring and deep water anchoring.

The data for the burial depth of submarine pipelines was fitted and analyzed for both anchor-dropping methods, as shown in Fig 4.

From Fig 4, certain rules can be defined relating to the effective burial depth of the representative ship type with different anchor weights:

1. For ordinary anchoring operations of the same representative ship type, as the water depth increases, the required effective burial depth of the submarine pipelines also increases slowly.
2. When the water depth remains the same, as the anchor weight of the representative ship type increases, the required effective burial depth of the pipeline also increases rapidly.
3. For the same representative ship type, the impact of deep anchoring on the burial depth of submarine pipelines is less than that of ordinary anchoring.
4. The relationship between the burial depth of the submarine pipelines and the anchor weight of ship is approximately linear: the greater the anchor weight, the greater the burial depth, resulting in safer operation.
5. For both anchoring methods: when the anchor weight of the ship is between 3 and 8 tons, the required effective burial depth of the submarine pipelines is between 1 and 2.2 m; when the anchor weight of the ship is between 8 and 12 tons, the required effective burial depth of the submarine pipelines is between 1.9 and 3.3 m; when the anchor weight of the ship is between 12 and 16 tons, the required effective burial depth of the submarine pipelines is between 2.8 and 4.3 m; when the anchor weight of the ship is between 16 and 20 tons, the required effective burial depth of the submarine pipelines is between 3.5 and 5.2 m.

### Further discussion with reference to a real-life accident

At 1338 on March 12, 2009, a cargo ship bound from Shanghai to Caofeidian went out of control within the operating area of the southwest Bohai Sea oilfield. The captain ordered the emergency broke down without consideration of the submarine pipelines in the navigation area, thus the anchor had a huge penetration impact on the submarine oil and gas pipeline, resulting in the submarine pipeline rupturing and oil spillages between Wellhead Platform Bozhong 13–1 and Qikou 18–1 19 km (38°34.271'N, 118°51.068'E). The ship was a 5000WDT bulk carrier, with a length of 223 m, breadth 32.3 m, moulded depth of 17.9 m, full load draught of 12.8 m and ship draft of 11.8m.The Hall anchor parameters were as shown in Table 4.Thesubmarine pipelines had a diameter of 273.1mm, a wall thickness of 11.1mm and a burial depth of 1.5m.Sediment backfilling was used in the pipelines, with an effective volume-weight per unit of the filling material of 11×10^3^ *kg/m*^*3*^, giving a depth of the pipeline area of 20 m.

**Table 4:** Parameters of the ship’s Hall anchor.

Mass of anchor (kg)	h (mm)	B1 (mm)	H1 (mm)	L (mm)	H (mm)
8300	1788	1176	510	2545	3363

Using the general anchoring model described above, the following calculations were performed: a 50000DWT bulk carrier with an anchor mass of 8300kg will produce 360461 Joules of impact energy when anchoring at a depth of 20 m, and thus requires a safety burial depth of 2.28 m when the anchor point is at the bottom of existing submarine oil pipelines. Since the depth of the buried pipeline was only 1.5m, which is less than our calculated minimum depth of 2.28 m, the anchor hit the pipeline and caused the pipeline to rupture, resulting in spillage of a large volume of crude oil, causing nearby marine environmental pollution.

## Conclusions

In our discussion of a real-life accident, the designed burial depth of the pipeline was only 1.5m, and thus did not meet the required buried depth of 2.28m, proving that our model is reasonable.

When an anchor is freely released, its motion in the water may not show uniform acceleration. The release height of the anchor and the water depth do not have a great influence on the impact energy of the anchor. When the water is deep enough, the anchors soon reach force balance under viscous resistance and buoyancy. The anchor weight has been found to be the largest influencing factor on the required burial depth of the submarine pipelines. When an anchor of typical weight (4–12 tons) hits submarine pipelines, the protective layer requires a thickness within the range of 1–3m. However, when a large anchor (more than 20 tons) hits, the required thickness of the burial protective layer should be above 5m, since this thickness will ensure that the layer will effectively and thoroughly absorb the generated energy. When the water depth exceeds 25m, the deep water anchoring operation has a relatively lower impact on the required burial depth of the submarine pipelines compared with the ordinary anchoring method, although still having a serious impact.

## Supporting Information

S1 File Parameter symbol. (DOCX)
