# Supplementary material for: Influence of Anchoring on Burial Depth of Submarine Pipelines
Source: PLoS One. 2016 May 11;11(5):e0154954. doi: 10.1371/journal.pone.0154954 (PMC4864321; doi:10.1371/journal.pone.0154954)
Supplement: S1 File — (DOCX) [file pone.0154954.s001.docx]

*A_p_* impact area of submarine pipelines, *J/m^2^*;

*B* buoyant force of the anchor, *N*;

B width of the anchor bottom;

B1 width of the anchor arm;

*D* diameter of submarine pipelines, *m*;

E_p_ energy absorbed by the filling material, *J*;

*f* viscous resistance of the anchor, *N*;

*g* gravitational acceleration, *m/s^2^*;

*h_1_* height of the anchor-dropping position above the water surface, *m*;

*h_2_* height of water surface above the bottom of the water, *m*;

*h_3_* depth that the anchor penetrates into the seabed, *m;*

*h* length of fluke;

*H* length of the anchor arm;

*H1* length of anchor shackle;

*k* ratio coefficient, *kg/s*;

*L* length of the anchor bottom;

*m* weight of the anchor, *kg*;

*N_r_* bearing capacity coefficient of the filling material;

*N_q_* bearing capacity coefficient of the filling material;

*Q* energy of the impact on the submarine pipeline,*J*;

*r* effective radius of the anchor, *m*;

$r'$ effective volume-weight of the filling material per unit weight, *kg/m^3^*;

*T* The falling time of anchor reaches the bottom, s;

*t* The falling time of anchor, *s*;

*v* velocity of the dropping anchor, *m/s*;

*v_0_* initial velocity of the dropped anchor, *m/s*;

*v_1_* velocity of the anchor when touching the water, *m/s*;

*v_2_* anchor velocity when it reaches the bottom, *m/s*;

*V* volume of the anchor, *m^3^*;

*z* penetration depth of the falling object through the protection layer, *m*; $\eta$ viscosity coefficient of water;

$\rho$ mass density of the sea water, *kg/m^3^*;

$\lambda$ the length-width ratio of the anchor bottom
